# Inter-Specific Differences in Numerical Abilities Among Teleost Fish

**DOI:** 10.3389/fpsyg.2012.00483

**Published:** 2012-11-08

**Authors:** Christian Agrillo, Maria Elena Miletto Petrazzini, Christian Tagliapietra, Angelo Bisazza

**Affiliations:** ^1^Department of General Psychology, University of PadovaPadova, Italy

**Keywords:** *Xenotoca eiseni*, *Poecilia reticulata*, *Danio rerio*, *Betta splendens*, *Pterophyllum scalare*, Fish cognition

## Abstract

Adults, infants and non-human primates are thought to possess similar non-verbal numerical systems, but there is considerable debate regarding whether all vertebrates share the same numerical abilities. Despite an abundance of studies, cross-species comparison remains difficult because the methodology employed and the context of species examination vary considerably across studies. To fill this gap, we used the same procedure, stimuli, and numerical contrasts to compare quantity abilities of five teleost fish: redtail splitfin, guppies, zebrafish, Siamese fighting fish, and angelfish. Subjects were trained to discriminate between two sets of geometrical figures using a food reward. Fish initially were trained on an easy numerical ratio (5 vs. 10 and 6 vs. 12). Once they reached the learning criterion, they were subjected to non-reinforced probe trials in which the set size was constant but numerical ratios varied (8 vs. 12 and 9 vs. 12). They also were subjected to probe trials in which the ratio was constant, but the total set size was increased (25 vs. 50) or decreased (2 vs. 4). Overall, fish generalized to numerosities with a 0.67 ratio, but failed with a 0.75 ratio; they generalized to a smaller set size, but not to a larger one. Only minor differences were observed among the five species. However, in one species, zebrafish, the proportion of individuals reaching the learning criterion was much smaller than in the others. In a control experiment, zebrafish showed a similar lower performance in shape discrimination, suggesting that the observed difference resulted from the zebrafish’s difficulty in learning this procedure rather than from a cross-species variation in the numerical domain.

## Introduction

Though numerical abilities were once considered a unique human ability, numerous studies have now shown that other primates display the capacity to add, subtract, and order numerical information (Brannon and Terrace, 1998; Beran, 2004; Matsuzawa, 2009). The evidence collected in cognitive, developmental, and comparative research has led several authors to propose that adults prevented from verbal counting, infants and non-human primates possess similar numerical systems that are independent from language and culture (Feigenson et al., 2004; Hauser and Spelke, 2004; Beran, 2008). For instance, the performance of rhesus monkeys adheres to that of adult humans in two comparative studies where both species were presented identical stimuli (Cantlon and Brannon, 2006, 2007a). In chimpanzees, error rates and reaction times are constant in the subitizing range (1–4) while they increase monotonically for larger numbers, suggesting the existence of a subitizing-like mechanism in apes (Tomonaga and Matsuzawa, 2002).

Following the discovery of the remarkable numerical skills of primates, researchers initially believed in the existence of a sharp discontinuity in cognitive abilities between primates and other animal species. However, during the last decade, the presence of basic quantity abilities has been reported in other mammals (bears: Vonk and Beran, 2012; elephants: Perdue et al., 2012; dogs: West and Young, 2002; dolphins: Kilian et al., 2003), in birds (parrots: Pepperberg, 2006; Al Aïn et al., 2009; pigeons: Roberts, 2010), in fish (mosquitofish: Agrillo et al., 2009; angelfish: Gómez-Laplaza and Gerlai, 2011a,b; swordtails: Buckingham et al., 2007), and even in invertebrates (ants: Reznikova and Ryabko, 2011; bees: Gross et al., 2009; beetles: Carazo et al., 2009).

Such new evidence has prompted a debate as to whether or not all species share the same quantity systems. Some studies have reported interesting similarities between primates and non-primate species. For instance, different mammals (Ward and Smuts, 2007; Perdue et al., 2012), birds (Al Aïn et al., 2009), amphibians (Krusche et al., 2010), and fish (Gómez-Laplaza and Gerlai, 2011a) are affected by the numerical ratio when required to compare numerosities, as commonly reported in primates (Beran, 2004; Cantlon and Brannon, 2007a). Rhesus monkeys can discriminate 1 vs. 2, 2 vs. 3, and 3 vs. 4, but not 4 vs. 5 (Hauser et al., 2000), the same limit exhibited by distantly related species, such as Eastern mosquitofish (Agrillo et al., 2008), guppies (Agrillo et al., 2012a), and robins (Hunt et al., 2008). Domestic chicks and robins can make spontaneous use of numerical information instead of using non-numerical perceptual cues that co-vary with number, such as cumulative surface area or density (Hunt et al., 2008; Rugani et al., 2009), which aligns with what has been reported in human (Cordes and Brannon, 2008; Nys and Content, 2012) and non-human primates (Cantlon and Brannon, 2007b). Similar performance in the discrimination of small and large numbers recently has been reported in a study comparing humans and guppies (Agrillo et al., 2012a).

However, many other studies have evidenced that performance varies across species in many respects. For example – unlike primates, chicks and robins – cats and dolphins seem to use numerical information only as a “last-resort” strategy, when no other continuous information is available (Kilian et al., 2003; Pisa and Agrillo, 2009). Horses, chicks, salamanders, and angelfish seem to be able to discriminate between groups differing by one unit up to 2 vs. 3 items (Uller et al., 2003; Rugani et al., 2007; Uller and Lewis, 2009; Gómez-Laplaza and Gerlai, 2011b), while mosquitofish, guppies, and robins discriminate up to 3 vs. 4 items (Agrillo et al., 2008, 2012a; Hunt et al., 2008). Trained pigeons can discriminate up to 6 vs. 7 items (Emmerton and Delius, 1993), well above the limit of number discrimination of 2 vs. 3 items observed in trained chicks (Rugani et al., 2007). Differences have been reported even between closely related species. For example, the ability to discriminate between large quantities appears to be affected by numerical ratio in African elephants (Perdue et al., 2012), but not in Asian elephants (Irie-Sugimoto et al., 2009).

Despite the wealth of comparative data, cross-species comparison has been difficult because the tasks investigated, the methodology employed, the sensory modality involved, and the context of species investigation vary considerably from one study to another. In some cases, the inconsistency is clearly to be ascribed to the different methods adopted. For example, when required to discriminate 1 vs. 2 and 2 vs. 3, the numerical ratio plays a key role in infants’ performance using auditory stimuli (vanMarle and Wynn, 2009), but not visual stimuli (Feigenson et al., 2002). Similarly, in goldbelly topminnows, the performance in a quantity discrimination task was affected by the type of procedure, with fish able to discriminate 2 vs. 3 only in one of two different procedures (Agrillo and Dadda, 2007).

To date, cross-species comparison using the same methodology rarely has been attempted; such studies have related exclusively to primates (Cantlon and Brannon, 2006, 2007a; Hanus and Call, 2007). To fill this gap, the present study compares the numerical abilities of five teleost fish (redtail splitfin, guppies, zebrafish, Siamese fighting fish, and angelfish) using the same procedure, stimuli, and numerical contrasts. Subjects were trained with a food reward to discriminate between two sets of geometrical figures differing in numerosity. Fish initially were trained on an easy numerical ratio (0.50). Once they reached the learning criterion, they were tested in non-reinforced probe trials for their ability to generalize to more difficult ratios (0.67 and 0.75), or to a larger or a smaller total set size. In addition, because the proportion of individuals reaching the initial learning criterion was very low in one species, we conducted a control experiment on shape discrimination to assess if this difference was specific to the numerical domain or was due to a more general cross-species difference in learning with this procedure.

## Experiment 1

### Subjects

The initial subjects of this experiment were 16 *Xenotoca eiseni* (redtail splitfin, total length: mean ± SD: 3.02 ± 0.25 cm), 16 *Poecilia reticulata* (guppies, 2.01 ± 0.30), 16 *Betta splendens* (Siamese fighting fish, 3.36 ± 0.32), 16 *Pterophyllum scalare* (angelfish, 4.09 ± 0.38), and 16 *Danio rerio* (zebrafish, 2.84 ± 0.27). All subjects were adult females with the exception of the group of angelfish composed by unsexed juvenile individuals. Redtail splitfin came from the stocks maintained in our lab; guppies were also maintained in our lab and were fifth generation descendants of wild-caught fish from the Tacarigua River in Trinidad. Siamese fighting fish, angelfish, and zebrafish were obtained from local commercial suppliers. As few zebrafish reached the criterion, we increased the sample size for this species by adding 10 more specimens of the same strain (hereafter called “commercial stock”) and by testing 22 more specimens from another strain coming from the outbreed stock maintained at the Biology Department of University of Padua (hereafter called “lab stock”).

Subjects were stocked at the Laboratory of Comparative Psychology (University of Padua) for at least 15 days before the beginning of the experiments and maintained in 150 l stock aquaria; each species was housed separately. Aquaria were provided with natural gravel, air filters, and live plants. Both stock aquaria and experimental tanks were maintained at a constant temperature of 25 ± 1°C and a 14:10 h light:dark (L:D) photoperiod; stock aquaria were lit by an 18-W fluorescent light, experimental tanks were lit by two 36 W fluorescent lamps. Before the experiment, fish were fed twice daily to satiation with commercial food flakes and live brine shrimp (*Artemia salina*).

### Apparatus and stimuli

**Figure 1:**
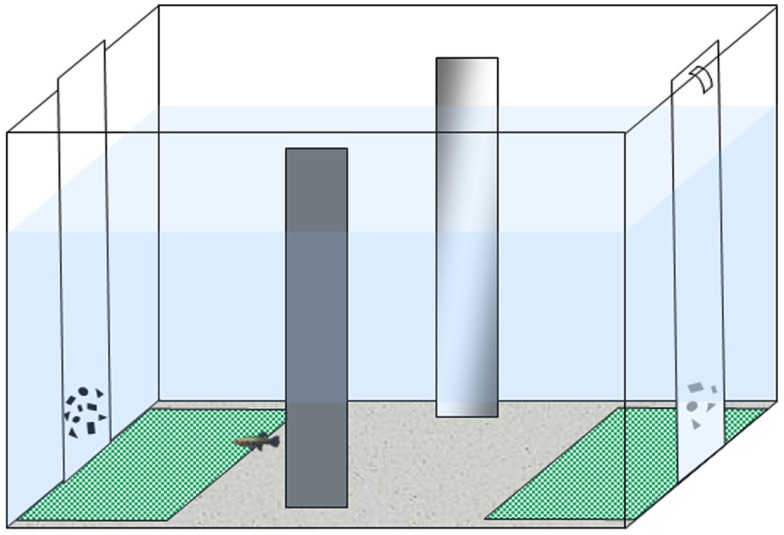
**Experimental apparatus**. Subjects were housed in the experimental tank for the entire experiment. Stimuli (two groups of geometric figures differing in numerosity) were presented at the bottoms of the tank.

We followed the apparatus and procedure described in a recent study (Agrillo et al., 2012b). The experimental apparatus was composed of a 50 cm × 19 cm × 32 cm tank (Figure 1) filled with gravel and 24 cm of water. The long walls were covered with green plastic material, while the short ones were covered with white plastic material. Two mirrors (29 cm × 5 cm) were placed in the middle of the tank, 3 cm away from the long walls, in order to reduce the potential effects of social isolation (Miletto Petrazzini et al., 2012). In addition, an artificial leaf (9 cm × 8 cm) was placed in the middle to provide some shelter for the subjects. In correspondence with the sides in which stimuli were presented, two “choice areas” were defined by white rectangles (14 cm × 12 cm) covered by a green net.

**Figure 2:**
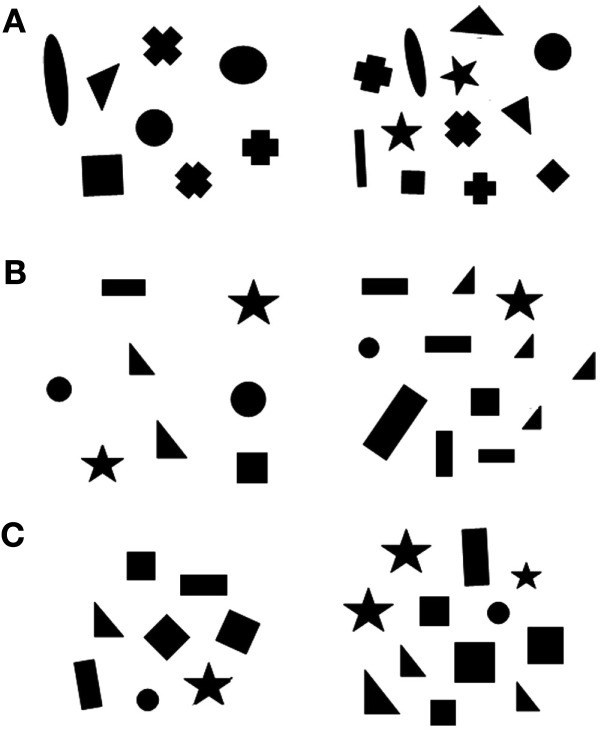
**Schematic representation of the stimuli used**. Each panel contained black geometric figures differing in numerosity inserted in a white background. In the figure we depicted a 8 vs. 12 contrast with cumulative surface area controlled to 100% **(A)**, to 85% **(B)** and to 70% **(C)**. In **(A)** and **(B)** stimuli are controlled for overall space, whereas in **(C)** they are controlled for density.

Stimuli were inserted in a 6 cm × 6 cm square and were presented at the bottom of a 6 × 32 transparent plexiglass panel. There were groups of black geometric figures differing in size on a white background. We presented different numerical contrasts: 5 vs. 10 and 6 vs. 12 (0.50 ratio) in the training phase; 8 vs. 12 and 9 vs. 12 (0.67 and 0.75 ratios) in phase 1; 2 vs. 4 and 25 vs. 50 in phase 2. Stimuli selected for the experiment were extracted from a pool of 24 different pairs for each numerical contrast. Both the size and position of the figures were changed across sets to avoid the fish having to discriminate the overall configuration of the stimuli instead of using numerical information. In addition, it is known that numerosity co-varies with other physical attributes, such as cumulative surface area, overall space occupied by sets, or density of the elements; human and non-human animals can use these non-numerical cues to estimate which group is larger/smaller (Pisa and Agrillo, 2009; Gebuis and Reynvoet, 2012). Cumulative surface area was controlled to reduce the possibility of fish using non-numerical cues. In particular, for one-third of the stimuli, the two numerosities were equated for cumulative surface area (100%). However, as a by-product of equaling cumulative surface area, smaller than average figures were more frequent in the larger groups, and fish could have used this cue instead of numerical information. To reduce this possibility, in another third of the stimuli, cumulative surface area was controlled to 85%, and, in a final third of the stimuli, it was controlled to 70% (Figure 2). In addition, since density and overall space encompassed by the stimuli are negatively correlated, half of the sets was controlled for density, whereas the second half was controlled for overall space. Cumulative surface area, density and overall space represent the most non-numerical continuous variables controlled in numerical cognition studies (Vos et al., 1988; Durgin, 1995; Kilian et al., 2003; Pisa and Agrillo, 2009). They also represent the only cues that proved to be sometimes used by fish with static stimuli (Agrillo et al., 2008, 2009, 2010; Gómez-Laplaza and Gerlai, 2011b).

Sixteen identical experimental tanks were used. They were placed close to each other on the same table. A video camera was suspended about 1 m above the experimental tanks and used to record the position of the subjects during the tests.

### Procedure

The experiment was divided into three different steps: (a) pre-training, (b) training, and (c) test.

Pre-training (a) was set up to permit fish to familiarize themselves with the experimental apparatus. Subsequently (b), all fish were singly trained to discriminate an easy numerical ratio (0.50) with the purpose of selecting those fish that successfully accomplished the numerical task.

Fish who reached the criterion were subsequently tested with novel numerical contrasts (c). This was divided in two phases: in phase 1, we observed the ability to discriminate between large numbers with two different numerical ratios: 8 vs. 12 (0.67) and 9 vs. 12 (0.75); in phase 2, we assessed whether fish showed the ability to generalize the numerical rule to novel numerosities having the same ratio (0.50), but very different total set size (2 vs. 4 and 25 vs. 50).

#### Pre-training

During the 6 days preceding the beginning of training, fish gradually were familiarized with the apparatus. On days 1–2, groups of 4 subjects of the same species were inserted in the experimental apparatus for a total of 4 h; on days 3–4, two subjects of each species were inserted in the apparatus for 4 h, while on days 5–6, fish stayed singly in the apparatus for the whole day. During this latter period, fish were fed twice a day. *Artemia* nauplii (*A. salina*) were inserted in the morning and in the afternoon near the two short walls.

Siamese fighting fish are known to be poorly social, as a consequence they were the only exception to this procedure. For this species, pre-training was identical but subjects were individually inserted in the apparatus starting from day 1.

#### Training

On days 7–9, fish received four trials per day (three consecutive days, for a total of 12 trials). Each trial consisted of inserting the two stimuli panels on the short walls. Two numerical contrasts were presented in a pseudo-random sequence: 5 vs. 10 and 6 vs. 12. Half of the fish were reinforced to the larger numerosities, while the second half was reinforced to the smaller numerosities. Soon after the stimuli were inserted into the tank, a Pasteur pipette was inserted to release the food reward (nauplii of *A. salina*) in correspondence with the reinforced numerosity; an identical syringe was simultaneously used to insert pure water close to the non-reinforced numerosity. Subjects were left free to feed for 7 min. After this time, stimuli were removed from the tank. The inter-trial interval lasted 3 h. We counterbalanced the position of the stimuli (left-right) over trials.

On days 10 and 11, two probe trials were alternated with two reinforced trials each day (four probe trials). In probe trials (two trials with 5 vs. 10 and two trials with 6 vs. 12, presented in a pseudo-random sequence), stimuli were inserted for 4 min in the tank without any reinforcement (extinction procedure). Pipettes were not inserted. The proportion of time spent in the “choice areas” was recorded as a measure of their capacity to discriminate the two numerosities. In particular subjects were considered as selecting a stimulus when their heads were inside the choice area associated to that stimulus. Reinforced trials were identical to those described for days 7–9. Only fish that met the criterion (defined as 60% of the time spent near the reinforced numerosity in probe trials) were selected for the test. A previous study (Agrillo et al., 2012b) has shown that, in easy tasks, such a criterion permits to distinguish fish that learn discriminations from those fish that continue to choose randomly. In a recent experiment we observed that fish that do not meet this criterion after the first 12 trials do not improve their performance even after extensive training (unpublished data).

Subjects were moved from one tank to another one at the end of each day in order to avoid the possibility of using the local/spatial cues of the tank. The latter was previously occupied by conspecific subjects.

#### Test

After a short interval (days 12–13) in which subjects received two reinforced trials each day with the same numerical contrasts presented during training, fish started the test. The test was composed of two different phases. In phase 1, three probe trials were presented each day for four consecutive days (days 14–17). Fish were presented with two different numerical ratios, 0.67 (8 vs. 12) and 0.75 (9 vs. 12), six presentations for each ratio in a pseudo-random sequence. The inter-trial interval lasted 3 h. Two reinforced trials presenting the numerical contrasts of the training (5 vs. 10 and 6 vs. 12) were alternated in the probe trials.

In phase 2, four probe trials were presented each day for two consecutive days (days 18–19). Fish were observed for their ability to generalize to small (2 vs. 4) and large (25 vs. 50) numbers; there were four presentations for each ratio in a pseudo-random sequence. The numerical ratio was equal to 0.50.

In both phase 1 and 2, we considered the proportion of time spent in the “choice areas” (accuracy) during probe trials as the dependent variable. Proportions were arcsine (square root)-transformed (Sokal and Rohlf, 1995). Mean ± SD are provided. Statistical tests were carried out using SPSS 18.0.

### Results

#### Training

In zebrafish, 5 out of 26 fish in the commercial stock and one out of 22 of the lab stock reached the criterion. The two strains of zebrafish did not differ in performance [independent *t*-test, *t*(46) = 1.48, *p* = 0.148] and were pooled together in subsequent analyses. A total of 42 fish reached the criterion and were admitted to the following phases (10 out of 16 redtail splitfin, 8/16 guppies, 10/16 Siamese fighting fish, 8/16 angelfish, and 6 out of 48 zebrafish). We found a significant difference among the species in the number of subjects reaching the criterion [chi square test, χ(4) = 23.48, *p* < 0.001]. This finding results from the fact that the number of individuals reaching the criterion in zebrafish was significantly lower compared to the other four species [zebrafish: 6/48, 12.5%; remaining four species: 36/64, 56.3%; chi square test, χ(1) = 22.4, *p* < 0.001].

No difference among the species was found in the proportion of time spent in the choice areas [one way ANOVA, *F*(4, 37) = 0.94, *p* = 0.452]. In particular, when analyzing the time spent in the choice areas of all individuals, no difference was found between zebrafish and the other species pooled together [independent *t*-test, *t*(110) = 0.84, *p* = 0.400].

#### Test

##### Phase 1: influence of numerical ratio

We found no difference in the accuracy between fish trained with the larger or smaller numerosities as positive (independent *t*-tests, *p* > 0.05 for both ratios).

**Figure 3:**
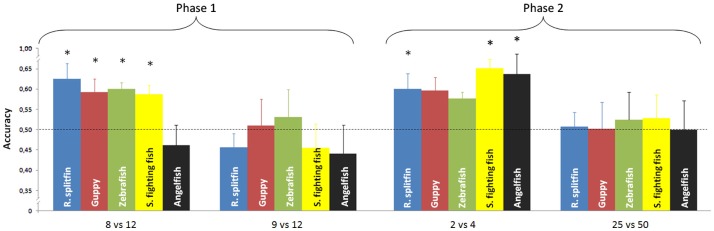
**Numerical contrasts were plotted against the accuracy of the five species**. Most of the species significantly discriminated 8 vs. 12 and spontaneously generalized to smaller numerosities (2 vs. 4). All species failed to discriminate 9 vs. 12 or generalize to larger numerosities (25 vs. 50). Asterisks denote a significant departure from chance level (*p* < 0.05). Bars represent the standard error.

No species proved able to discriminate 9 vs. 12 items (Table 1). There was no difference in performance among the five species [one way ANOVA, *F*(4, 37) = 0.45, *p* = 0.772]. All species, except angelfish, significantly discriminated 8 vs. 12 items (Table 1). A significant difference among the five species was found for this ratio [one way ANOVA *F*(4, 37) = 3.30, *p* = 0.021]. On the whole there was a significant difference between the two numerical ratios [repeated measure ANOVA, Ratio: *F*(1, 37) = 9.42, *p* = 0.004; species: *F*(4, 37) = 1.59, *p* = 0.197; interaction: *F*(4, 37) = 0.70, *p* = 0.597, Figure 3].

**Table 1:** **Performance of the five species in the numerical contrasts presented during test phase**.

Species	8 vs. 12	9 vs. 12	2 vs. 4	25 vs. 50
Redtail splitfin	*t*(9) = 3.25, *p* = 0.010*	*t*(9) = 1.26, *p* = 0.239	*t*(9) = 3.12, *p* = 0.012*	*t*(9) = 0.18, *p* = 0.861
Guppy	*t*(7) = 2.86, *p* = 0.024*	*t*(7) = 0.21, *p* = 0.842	*t*(7) = 2.02, *p* = 0.083	*t*(7) = 0.05, *p* = 0.961
Zebrafish	*t*(5) = 6.10, *p* = 0.002*	*t*(5) = 0.56, *p* = 0.597	*t*(5) = 1.55, *p* = 0.181	*t*(5) = 0.42, *p* = 0.690
Siamese fighting fish	*t*(9) = 3.95, *p* = 0.003*	*t*(9) = 0.79, *p* = 0.453	*t*(9) = 3.42, *p* = 0.008*	*t*(9) = 0.40, *p* = 0.698
Angelfish	*t*(7) = 0.79, *p* = 0.458	*t*(7) = 0.87, *p* = 0.414	*t*(7) = 2.50, *p* = 0.041*	*t*(7) = 0.76, *p* = 0.942

##### Phase 2: generalization to small and large numerosities

No species proved able to generalize the learned discrimination to a larger set size, 25 vs. 50 items (Table 1). There was no difference in performance among the five species [one way ANOVA, *F*(4, 37) = 0.06, *p* = 0.992]. Three species (redtail splitfin, Siamese fighting fish, and angelfish) significantly generalized the learned discrimination to a smaller set size, 2 vs. 4 items. One species (guppy) yielded a marginally significant result, and one species (zebrafish) was not significant (Table 1). However, the trend is similar in all five species, and the difference among them is not significant [one way ANOVA, *F*(4, 37) = 0.49, *p* = 0.741]. A likelihood ratio analysis (see Glover and Dixon, 2004 for details) confirmed that the probability that the five species do not differ is three times larger (λ = 2.98) than the probability that a difference exists. Overall, the difference in the generalization between the larger and smaller set size was significant [repeated measure ANOVA, *F*(1, 37) = 9.84, *p* = 0.003] with no species variation [*F*(4, 37) = 0.23, *p* = 0.919; interaction: *F*(4, 37) = 0.06, *p* = 0.911, Figure 3].

We found no difference in the accuracy between fish trained with the larger or smaller numerosities as positive [2 vs. 4, independent *t*-test, *t*(40) = 1.34, *p* = 0.187; 25 vs. 50, independent *t*-test, *t*(40) = 0.22, p = 0.826].

## Experiment 2

### Subjects, apparatus, stimuli, and procedure

Twenty fish (10 *D. rerio* and 10 *X. eiseni*) were tested. Both species were observed in a discrimination between two black geometric figures in a white background (filled triangle vs. empty circle). For each species, half of the subjects were reinforced to the triangle, and half to the circle. The same figures were presented during all trials (both training and probe trials). The apparatus was identical to that of experiment 1. The procedure also was the same, with the exception that the experiment ended after the four probe trials of the training phase.

### Results

**Figure 4:**
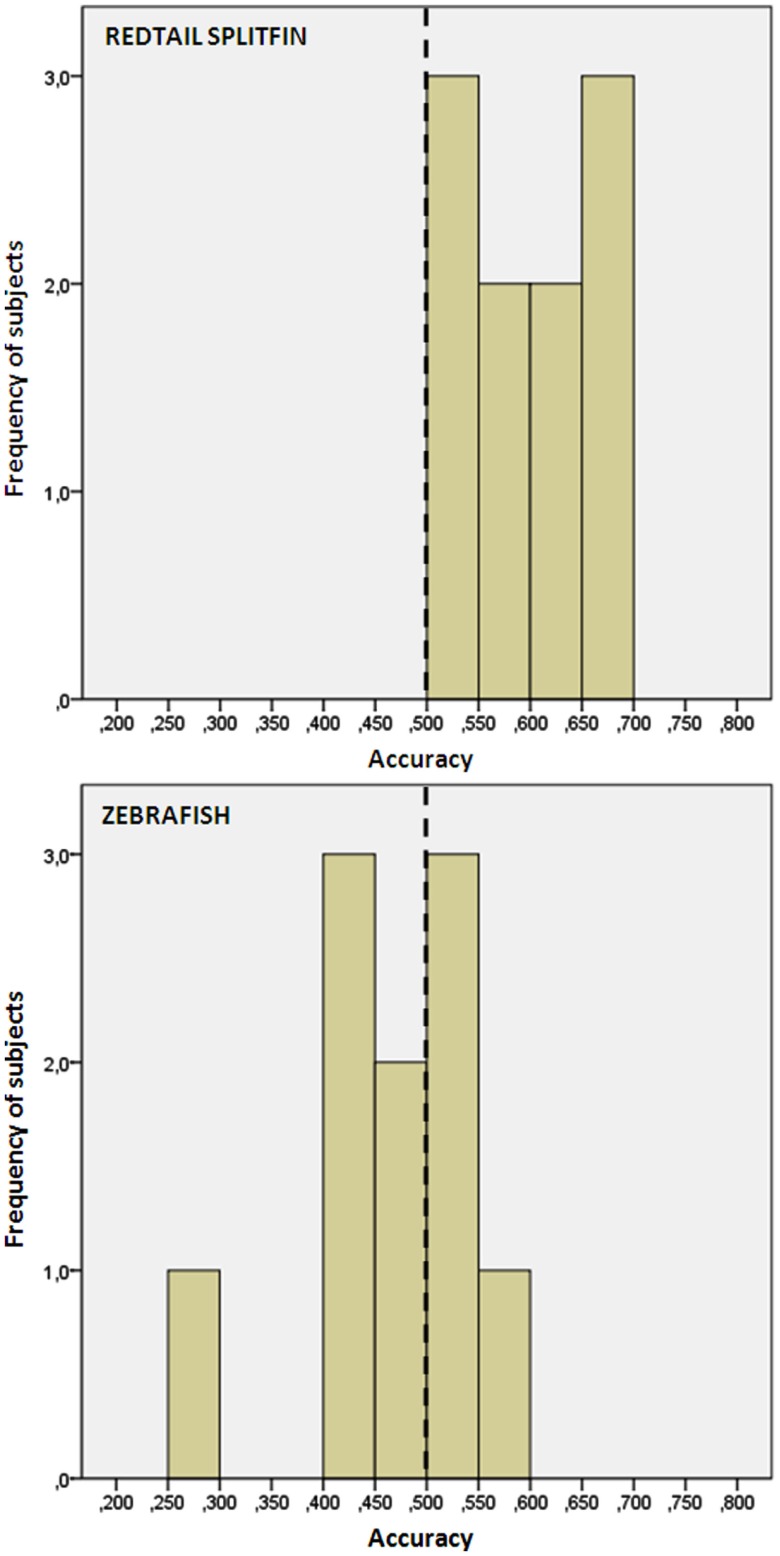
**Frequency distribution of the preference score of zebrafish and redtail splitfin in the shape discrimination task of experiment 2**. Redtail splitfin significantly preferred the reinforced stimulus, while the performance of zebrafish is indistinguishable from random choice.

We found no difference in the accuracy between fish trained with the triangle or circle as positive (independent *t*-tests, *p* > 0.05 for both species). Redtail splitfin significantly discriminated between the two figures [mean ± SD: 0.594 ± 0.06, one sample *t*-test, *t*(9) = 4.65, *p* = 0.001], while zebrafish did not [0.471 ± 0.08, one sample *t*-test, *t*(9) = 1.08, *p* = 0.307]. A significant difference between the two species was found [independent *t*-test, *t*(18) = 3.70, *p* = 0.002, Figure 4]. No difference in the accuracy was found between fish trained in numerical discrimination (training of phase 1) and those trained to discriminate geometric figures [independent *t*-test for unequal cases redtail splifin: *t*(24) = 0.807, *p* = 0.428; zebrafish: *t*(56) = 0.005, *p* = 0.996].

## Discussion

The present paper represents the first attempt to compare numerical abilities in teleost fish using the same methodology. Subjects of five teleost species first were trained to discriminate two sets of geometrical figures using an easy 0.50 numerical ratio (5 vs. 10 and 6 vs. 12) and then observed in non-reinforced probe trials in which the numerical ratios or total set size varied. Overall, similarities were far greater than differences. Fish trained with the larger or smaller numerosities as positive showed equal accuracy in all species. When we made the discrimination more difficult by increasing the numerical ratio, we observed a similar pattern of performance in all fish, with no species being able to discriminate the 0.75 ratio (9 vs. 12), but four out of five species being able to discriminate the 0.67 ratio (8 vs. 12). The pattern of generalization of the numerical rule to a different set size was also very similar in the different species. Fish generalized the learned discrimination to a smaller set size (2 vs. 4), showing no substantial inter-specific difference, while no species was able to generalize to a larger set size (25 vs. 50). These data, together with results recently reported in another teleost species tested in the same apparatus, *Gambusia holbrooki* (Agrillo et al., 2012b), point toward the existence of similar numerical discrimination among fish.

In all, we observed three main inter-specific differences. First, the proportion of subjects that reached the criterion in the training phase was similar among species, with the exception of zebrafish, which showed a fourfold smaller proportion of fish reaching the criterion. This difference might be ascribed to two reasons: (a) a specific deficit of zebrafish regarding numerical skills, or (b) a more general inability of this species in discrimination learning. The results of experiment 2 support the latter hypothesis. When trained to learn simple shape discrimination, a filled triangle from an empty circle, zebrafish performed much worse than the control species, redtail splitfin. In recent years, a few works have been published regarding the possibility of training visual discrimination in zebrafish. In most cases, the required discrimination was even more simple than this, i.e., to distinguish a red wall from other non-colored ones (Sison and Gerlai, 2010), or implied a much larger number of trials (Braubach et al., 2009). To our knowledge, there are no data that allow a direct comparison between zebrafish and other teleosts in the same procedure. It therefore remains to be seen whether the difference between zebrafish and other species is specific to the method we used in this study or extends to other learning tasks. It is important to note that the few zebrafish reaching the criterion were similar in performance to the other four species in both phase 1 and 2, reinforcing the hypothesis that the low performance of zebrafish primarily resulted from a low learning performance in this species.

Different learning performance might in turn be explained with species-specific characteristics, such as neophobia. Consistent differences in behavior between individuals in a population, especially in the shy-bold continuum, have been reported in a variety of organisms, including many fish species (Dall et al., 2004; Sih et al., 2004); it has been termed “animal personalities” or “coping styles.” In many conditions, these different coping styles may affect the speed of acquiring a task (Sneddon, 2003; Kurvers et al., 2010; Amy et al., 2012). One might argue for instance that a shy species may have explored the experimental tank less than a bold species, thus having less time to associate the proximity to the positive stimulus with food reinforcement. However, this is not the case in our experiment, as we found that the proportion of time spent in the two choice areas by zebrafish was the same as other species.

The second difference among the species was observed in phase 1. Unlike the other four species, angelfish seem to be unable to discriminate 8 vs. 12. Such a result is puzzling and even surprising if we consider that angelfish tested with another paradigm (spontaneous shoal choice) showed the same or even better performance than mosquitofish and guppies in large number discrimination (Agrillo et al., 2008, 2012a; Gómez-Laplaza and Gerlai, 2011a). *P. scalare* is larger species and, in order to match as far as possible the five species in size, we had to test sub-adult angelfish. This factor could potentially account the differences observed in this species. However we believe this is an unlikely explanation for the differences observed in this experiment, as other studies have shown that numerical abilities of very young fish are not much dissimilar from those observed in the adults (Bisazza et al., 2010). It is worth noting that, although the subjects of the five species had comparable body length, the morphological characteristics of angelfish differ from those of the other species tested: in angelfish the longitudinal axis is shortened, and the body is laterally compressed with extended dorsal and anal fins and we cannot exclude that water depth used in experimental tanks was not entirely suitable for this species.

The difficulty to understand the exact nature of angelfish peculiarity highlights one of the main problems of comparative studies: the strength of using the same methodology for testing different species may become a methodological weakness. Different species show different adaptations to their different ecological niches and, therefore, housing and testing requirements could be different in the lab; some species might be affected by such daily handling more than some others, or have perceptual or motivational characteristics that potentially render the tests more dissimilar across different species than initially planned. To assess whether the apparent inability of angelfish to discriminate the 0.67 ratio is simply an artifact of the methodology adopted, replication studies using different methods are needed (Agrillo and Miletto Petrazzini, 2012).

A third possible inter-specific difference was observed in phase 2 in which the generalization to small numbers was fully significant only for three species. The trend is, however, similar for the five species, and the likelihood ratio analyses indicated that the lack of difference among the species was 2.98 times more likely than the alternative hypothesis. One may argue that results of phase 2 might be affected by potential carry-over effects from phase 1, as all subjects performed the experiments in the same order (generalization to different numerical ratios firstly and generalization to different total numerosities secondly). However, it is worth noting that in the whole experiment 1 fish were trained only in a 0.50 ratio and were exposed to more difficult ratios without receiving any reinforcement.

**Figure 5:**
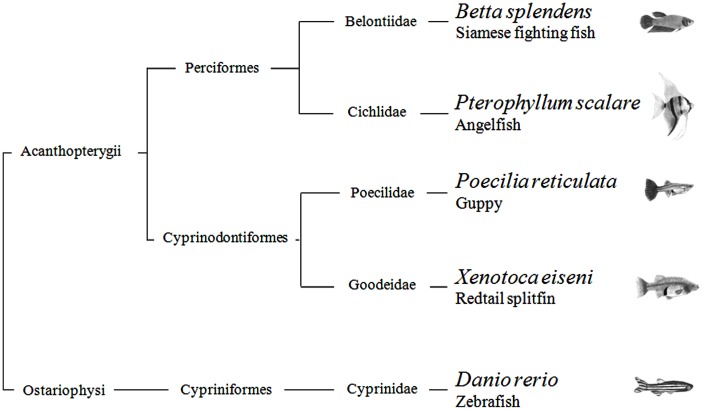
**Phylogenetic relationship of the five teleost species used in the study**.

In summary, with the possible exception of the angelfish results in one of four generalization tests, this study provides scarce evidence that quantification systems differ across teleosts. There is current debate regarding non-verbal numerical systems. Some scholars argue that they are the same in all vertebrates, inherited from a common ancestor; others believe that each species has evolved its mechanisms in relation to the constraints imposed by the nervous system and the ecological problems faced in the environment. From a phylogenetic point of view, the five species studied here are distantly related. According to recent estimates, the Ostariophysi (Figure 5), the group to which zebrafish belongs, and the Acanthopterygii, the group which comprises the other four species, diverged more than 250 million years ago (Steinke et al., 2006). They also encompass a broad spectrum of ecological adaptations. For example, some species live in open areas and others densely vegetated shallow waters, some are highly gregarious and other basically solitary, some care their young and other provide no form of parental care. The finding of so few inter-specific differences seems more in accord with the existence of ancient quantification systems inherited from a common ancestor. On the other hand, the species have been compared in a single context, and they may reveal larger differences if studied in wider spectrum of domains.

The observation made in this study that fish can easily generalize to sets of reduced numerosity but not to sets of enlarged numerosity deserves consideration. The failure to generalize the numerical rule learned in 5 vs. 10 and 6 vs. 12 in contrast to 25 vs. 50 items is particularly surprising considering that previous studies (Agrillo et al., 2010, 2012b) showed that mosquitofish can successfully generalize to even larger numerosities, such as 100 vs. 200, provided that they receive some reinforced trials on these new stimuli. One could argue that during training, fish may have learned to choose a precise numerosity instead of learning a number rule (i.e., choose the larger/smaller quantity), and later they preferred the quantity closest to that previously reinforced. For example, a fish trained to choose 12 over 6 items during 25 vs. 50 probe trials might prefer 25 items because it is the closest to the reinforced numerosity. In this case, however, we would expect the same phenomenon to occur during the generalization to smaller numerosities, which did not happen. In addition, we expect an opposite performance depending on whether fish have been trained toward the smaller or the larger numerosity. Yet, no difference was found between these two conditions, thus excluding the possibility that the direction of the training might have interfered with the direction of the variation of total set size.

We can only speculate about the meaning of this result. In nature, some items in a set may partially hide each other or temporarily disappear behind objects, thus reducing the visible total set size even if their composition is constant. For example, during the comparison of 5 vs. 10 conspecifics, fish might be required to continue the enumerating process when the perceived numerosity is reduced, for example when only 4 vs. 8 fish are visible. In this sense, the cognitive systems of these species might have been selected to generalize the numerical rule to another contrast with a reduced total set size. In contrast, it is physically implausible that groups of objects increase their numerosity without altering their composition. In other words, while 2 vs. 4 would appear as another version of the 5 vs.10 task, the shift from 5 vs. 10 to 25 vs. 50 items might appear to fish as a novel task, preventing generalization of the same numerical rule from smaller to larger numbers. It will be a challenging task to determine whether other vertebrate species show the same generalization pattern.

As a last remark, we would like to note one important implication of the findings from experiment 2. While the results of the training phase in experiment 1 would superficially suggest cross-species differences in numerical abilities, the difference observed between redtail splitfin and zebrafish in another type of discrimination showed the true nature of zebrafish low performance. When investigating the existence of differences between experimental groups in one cognitive domain, it is always important to include control tests done in other domains to exclude the possibility that the observed differences depend on concomitant factors, such as personality, motivation, or attention differences. This is routinely performed in other disciplines (i.e., cognitive psychology), but still rarely adopted in comparative psychology studies.

## Conflict of Interest Statement

The authors declare that the research was conducted in the absence of any commercial or financial relationships that could be construed as a potential conflict of interest.
